# Editorial: News and Views in the Management of Myasthenia Gravis

**DOI:** 10.3389/fneur.2021.769086

**Published:** 2021-10-07

**Authors:** Amelia Evoli, Nils Erik Gilhus, Jeffrey Guptill

**Affiliations:** ^1^Dipartimento di Neuroscienze, Università Cattolica del Sacro Cuore, Roma, Italy; ^2^Policlinico Universitario A. Gemelli, Istituto di Ricovero e Cura a Carattere Scientifico, Roma, Italy; ^3^Department of Neurology, Haukeland University Hospital, Bergen, Norway; ^4^Department of Clinical Medicine, University of Bergen, Bergen, Norway; ^5^Department of Neurology, Duke University School of Medicine, Durham, NC, United States

**Keywords:** myasthenia gravis, acetylcholine receptor antibodies, muscle-specific tyrosine kinase antibodies, immunosuppressive therapy, quality of life measures

Myasthenia gravis (MG) is a rare disease of the neuromuscular transmission and one of the best characterized autoimmune diseases. The aim of this Research Topic was to provide an overview of current issues in the management of this disease.

In MG, pathogenic antibodies (Abs) bind to key components of the motor end-plate and cause morphological and functional alterations of the postsynaptic membrane leading to loss of acetylcholine receptors (AChRs) and impairment of neuromuscular transmission. The clinical hallmark is fatigable weakness of striated muscles with broad phenotypic variability. The AChR is the main antigen in MG, followed by the muscle-specific tyrosine kinase (MuSK) and the low-density lipoprotein-related protein 4 (LRP4). Specific Abs identify disease subtypes with distinctive pathogenic aspects, clinical features, and response to therapy. In addition, Abs to synaptic proteins (agrin and collagen Q) and muscle proteins like titin, the ryanodine receptor, Kv1.4 potassium channel, and cortactin can be found in MG patients. Patient subgrouping according to the Ab profile is considered a prerequisite to optimizing treatment (1). Two contributions are focused on the role of Abs in the immuno-pathogenesis and management of MG. Frykman et al. review the Ab effects at the neuromuscular junction and propose a useful algorithm for MG serological diagnosis. Lazaridis and Tzartos discuss recent advances in Ab testing and prospects for future innovative antigen-specific therapies.

Like other autoimmune diseases, the etiology of MG is multifactorial, including self-tolerance disruption, genetic predisposition, and environmental factors. An imbalance between antigen-specific CD4+ T helper cells and regulatory cells is thought to be crucial in promoting B cell activation and high-affinity Ab production (2). Wu et al. revise the evidence for the involvement of different subsets of regulatory cells in MG pathogenesis and discuss the difficulty in translating these findings into the heterogeneous MG population. Vitamin D has modulatory effects on both innate and adaptive immune responses (3). The study by Han et al., investigating the association of vitamin D receptor polymorphisms with MG in the Chinese Han population, reports an increased frequency of the rs731236 variant in adult AChR-negative patients. With reference to the increased frequency of autoimmune diseases in MG patients compared to healthy subjects, Li et al. focus on the rare association with primary Sjögren's syndrome.

The MG diagnosis may be challenging in individuals with isolated ocular symptoms (ocular MG-OMG) given the low positivity rates of serological testing and repetitive nerve stimulation. In their review, Wirth et al. summarize the evidence on sensitivity and specificity of repetitive ocular vestibular evoked myogenic potentials (roVEMPs), a promising new technique that can detect muscle fatigability through direct recording from extrinsic ocular muscles.

MG management takes into consideration weakness extension and severity, associated Abs, age at onset, and thymus pathology. Current treatment is based on the use, generally in combination, of cholinesterase inhibitors, corticosteroids, other immunosuppressants, and, in selected subgroups, thymectomy. Plasma exchange and high-dose intravenous Ig (IVIg) are used in deterioration phases or as periodic treatment in patients with refractory disease. Farrugia and Goodfellow provide a comprehensive overview of MG management in adult patients, including data from the authors' own experience. Three contributions investigate selected therapeutic options. Imai et al. compare the efficacy of different prednisolone regimens in the long-term course of MG. Fan et al. assess the therapeutic effect and safety profile of tacrolimus monotherapy in patients with ocular and generalized disease. Putko et al. report the results of a *post-hoc* analysis, based on an open-label prospective trial of subcutaneous Ig (SCIg), to evaluate the correlation of SCIg dosage and serum IgG levels with clinical response.

Several articles in this collection focus on subgroups of patients in whom MG management poses specific problems or is complicated by the rarity of the disease and lack of randomized controlled trials (RCTs). N.E. Gilhus reviews the potential risks for mother and child during pregnancy, delivery, and the postpartum/postnatal period. Heckmann and Marais describe the characteristics of MG in human immunodeficiency virus (HIV) infected persons and discuss safety concerns related to immunosuppressive therapy. Evoli and Iorio provide an overview of OMG epidemiology, rate of progression to generalized MG, clinical aspects, and treatment issues. Two contributions focus on juvenile MG (age at onset ≤ 18 years). O'Connell et al. discuss the current evidence on disease management, propose a treatment algorithm, and highlight controversial issues in diagnosis and treatment. The study by Popperud et al. investigates the long-term effects of early-life thymectomy on the immune system by measuring T cell subsets at different intervals after surgery. Two articles address MG with Abs to MuSK (MuSK-MG). Rodolico et al. review the disease epidemiology, clinical phenotypes, diagnostic challenges, and response to therapy. Zhang et al. examine MuSK-MG severity and prognosis in Northern China through a comparison with AChR-MG and AChR/MuSK negative (double seronegative—DSN) MG.

Although most patients respond satisfactorily to conventional therapy, drug-free remission is rare, chronic immunosuppression is generally required, and 10–15% of patients have refractory disease (4). Targeted immunotherapies, including B cell depletion, inhibition of complement activation, and increased IgG clearance through interference with the Fc neonatal receptor, are promising alternatives to conventional immunosuppression. Two contributions focus on therapeutic advances in MG. Menon et al. review the rationale for the use of novel agents in MG and the status of related RCTs. Mantegazza and Antozzi examine the unmet needs in MG treatment, discuss the potential advantages of the early use of biologic drugs, and the prospects for new therapeutic approaches. Among non-pharmacological interventions, there has been increasing awareness of the beneficial effects of an active lifestyle. O'Connor et al. discuss the difficulty in quantifying fatigue perception in MG patients and review the current evidence on physical activity and tailored exercise training in patients with stable disease.

Outcome measures, including disease-specific scales aimed at quantifying muscle weakness and self-perceived quality of life (QoL) are crucial to assess the response to treatment in clinical practice (5) and patients' satisfaction with disease control (6). Thomsen and Andersen revise the use of ordinal scales in recent RCTs, and highlight some drawbacks such as the limited correlation between muscle weakness and disability, a considerable floor effect in milder cases, and lack of data about the performance of the scales in different patient populations. Applying the short-form 36-item questionnaire for health survey to a large patient cohort, Szczudlik et al. show that together with symptom severity, age and employment status are among the main determinants of reduced QoL in MG. The steroid- and, in general, the immunosuppressive therapy-sparing effect has increasingly been used as a treatment end-point. This approach has a strong rationale, as immunosuppression tapering is an indirect measure of disease control and the burden of treatment-related side effects has a negative impact on QoL. Nowak et al. report the changes in patients' exposure to conventional immunosuppression during the open-label extension of the REGAIN trial, which investigated the efficacy and safety of eculizumab in refractory AChR-MG (7). Around 50% of patients could withdraw one immunosuppressant and most could taper other agents with sustained disease control.

Phenotypic variability, immunopathological heterogeneity, and symptom fluctuations all contribute to the complexity of MG management. In addition to established protocols for disease confirmation and treatment, new diagnostic techniques and more selective immunotherapies have become available of late. Clinicians must be aware of their advantages and limitations in order to optimize treatment. This Research Topic addresses a broad range of clinical issues and should contribute to reach this goal.

## Author Contributions

All authors listed have made a substantial, direct and intellectual contribution to the work, and approved it for publication.

## Conflict of Interest

The authors declare that the research was conducted in the absence of any commercial or financial relationships that could be construed as a potential conflict of interest.

## Publisher's Note

All claims expressed in this article are solely those of the authors and do not necessarily represent those of their affiliated organizations, or those of the publisher, the editors and the reviewers. Any product that may be evaluated in this article, or claim that may be made by its manufacturer, is not guaranteed or endorsed by the publisher.
